# Significance of the Monitoring Right Ventricular Echocardiographic Parameters in Patients with Hypertrophic Cardiomyopathy Undergoing Alcohol Septal Ablation—A Single-Center Experience

**DOI:** 10.3390/diagnostics15192509

**Published:** 2025-10-02

**Authors:** Tibor Poruban, Ingrid Schusterova, Dominik Pella, Jan Fedacko, Karolina Angela Sieradzka Uchnar, Barbora Sepesiova, Silvia Gurbalova

**Affiliations:** 1 East Slovak Institute of Cardiovascular Diseases, School of Medicine, Pavol Jozef Safarik University, 040 01 Kosice, Slovakia; ischusterova@gmail.com (I.S.); dominik.pella@gmail.com (D.P.); sieradzka.ina@gmail.com (K.A.S.U.); bsepesiova@vusch.sk (B.S.); siska.buzova@gmail.com (S.G.); 2 Department of Gerontology and Geriatrics, Pavol Jozef Safarik University, 040 01 Kosice, Slovakia; jan.fedacko@upjs.sk

**Keywords:** hypertrophic cardiomyopathy, echocardiography, alcohol septal ablation, right ventricle

## Abstract

**Background/Objectives:** This study aimed to investigate the association between right ventricular (RV) structure and function and established markers of alcohol septal ablation (ASA) efficacy in patients with hypertrophic cardiomyopathy (HCM). We hypothesized that RV characteristics may serve as predictors of left ventricular outflow tract gradient (LVOT_G_) in the early period following ASA. **Methods:** A retrospective analysis was performed in 50 HCM patients who underwent ASA. Correlations between echocardiographic RV parameters and standard indicators of ASA success were assessed at 3 months, 1 year, 3 years, and 5 years post-procedure. **Results:** Echocardiographic measurements of RV wall thickness (RVWT) at 3 months and 1 year after ASA showed significant correlations with maximum LVOT_G_ (*p* < 0.001), NYHA functional class, and left ventricular end-diastolic dimension (LV_D_) (both *p* < 0.01). At 3 and 5 years, these correlations were no longer statistically significant (*p* = ns). No associations were observed for other parameters. **Conclusions:** Echocardiographic assessment of RVWT may serve as an early predictor of subsequent LVOT_G_ development as soon as 3 months after ASA. RVWT could therefore provide an estimate of long-term treatment effects. Further studies are needed to confirm these findings.

## 1. Introduction

Hypertrophic cardiomyopathy (HCM) is a genetic myocardial disorder defined by unexplained thickening of the left ventricular (LV) wall, most commonly due to pathogenic variants in sarcomeric protein genes, although a substantial proportion of cases lack an identifiable genetic mutation. The disease exhibits marked heterogeneity in its clinical presentation, with considerable variation in the degree and distribution of LV hypertrophy, as well as associated structural abnormalities such as mitral valve elongation and papillary muscle anomalies. HCM is associated with an increased risk of sudden cardiac death, atrial fibrillation, thromboembolic complications, and progressive heart failure, and its prevalence is estimated at approximately 1 in 500 adults worldwide [1,2,3].

Diagnosis is established by imaging—typically echocardiography or cardiac magnetic resonance—demonstrating a maximal LV wall thickness of ≥15 mm in any segment, or ≥13 mm in individuals with a family history of HCM and no alternative cause for hypertrophy [4,5]. Right ventricular involvement, while less common, can occur and may influence prognosis. HCM is classified as obstructive or non-obstructive based on the presence of dynamic LV outflow tract obstruction; the obstructive form, defined by a peak LV outflow tract gradient of ≥30 mmHg, is present in roughly two-thirds of patients, with obstruction occurring either at rest or only with provocation [6].

Obstruction is typically due to asymmetric septal hypertrophy, systolic anterior motion of the mitral valve, and papillary muscle abnormalities, and is a major contributor to symptoms such as exertional dyspnea, chest pain, palpitations, syncope, and sudden cardiac death [7].

**Figure 1:**
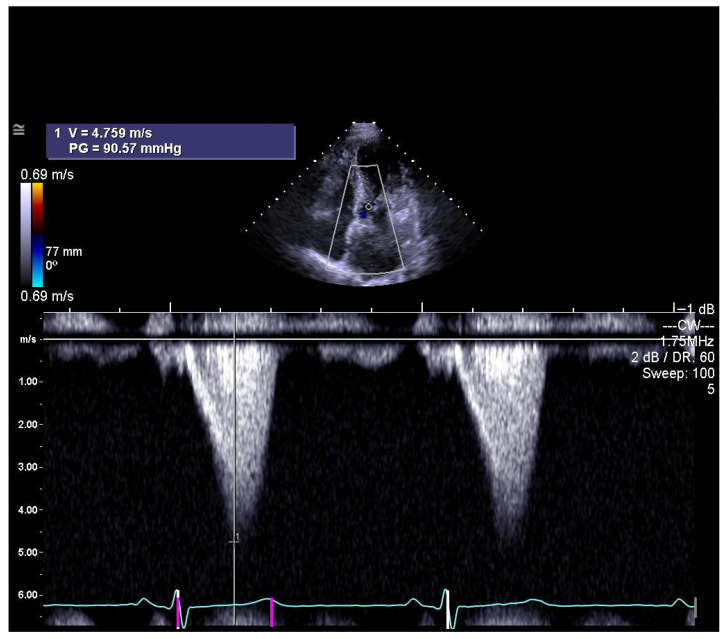
The left ventricular outflow tract (LVOT) shows a maximum gradient, usually with a late systolic peak, as assessed by continuous-wave Doppler echocardiography (photo and copyright: T.P.). PG—peak aortic valve gradient; V—maximum aortic valve velocity.

Global guidelines emphasize that septal reduction therapy (SRT) should only be performed in high-volume, specialized centers with experienced multidisciplinary heart teams to ensure the best outcomes. There is strong consensus regarding its indications in patients with obstructive hypertrophic cardiomyopathy. SRT is reserved for individuals who continue to experience significant symptoms—most often NYHA Class III–IV limitations such as severe shortness of breath or angina—despite optimal or maximally tolerated medical therapy. Across all recommendations, a peak left ventricular outflow tract (LVOT) gradient of ≥50 mmHg, whether at rest or during physiologic provocation, is considered essential (Figure 1). Additional requirements include systolic anterior motion (SAM) of the mitral valve and sufficient anterior septal thickness to allow safe procedural intervention. When determining the specific modality of SRT, guidelines consistently prefer surgical septal myectomy, especially for patients who also need surgical correction of other cardiac conditions. In cases where surgery is contraindicated or carries excessive risk, alcohol septal ablation (ASA) is endorsed as the alternative [1,2].

ASA was first performed by Sigwart in 1994 [8]. It involves the occlusion of the first septal branch of the left coronary artery and the subsequent application of absolute alcohol (1.0–3.8 mL) via catheter technique, inducing necrosis in this area. This results in the formation of scar tissue, which thins the previously hypertrophic IVS, leading to LVOT widening, reduction in its gradient, decrease in intraventricular pressure, and improvement in clinical condition [9].

Previous studies have presented the importance of determining RV wall thickness in the prognosis of HCM patients. However, to our knowledge, no article has yet been published evaluating the significance of RV echocardiographic parameters in patients undergoing ASA.

For this reason, the aim of this study was to examine the association between RV parameters and standard measures of success following ASA in patients with HCM. Our primary hypothesis was that the composition and function of the RV could predict the magnitude of LVOT_G_ in the early post-ablation period.

## 2. Materials and Methods

### 2.1. Study Population

The cohort included 50 young adult patients (27 men and 23 women) aged 60.8 ± 15.4 years, who underwent ASA for persistent limiting symptoms despite maximized medication therapy. Inclusion criteria were 1. echocardiographic image of HCM with obstruction in the LVOT and left ventricular ejection fraction (LVEF) ≥50%; 2. invasively measured resting LVOT gradient (LVOTG) ≥30 mmHg; 3. New York Heart Association (NYHA) classification >2; and 4. echocardiographic and clinical examination performed at 3 and 12 months after ASA. Exclusion criteria included poorly controlled arterial hypertension, severe aortic stenosis, known rare or infiltrative disease, implantation of an implantable cardioverter-defibrillator or dual-chamber pacemaker prior to the study.

All procedures and experimental protocols complied with the ethical standards of the institutional review board/ethics review committee (IRB/ERC) of the East Slovak Institute of Cardiovascular Diseases, Košice, Slovakia, and with the principles of the Helsinki Declaration (1975, revised 2008). Approval was granted on 17 May 2021. Written informed consent was obtained from all participants. The study’s principal investigator was T.P.

### 2.2. Alcohol Septal Ablation

**Figure 2:**
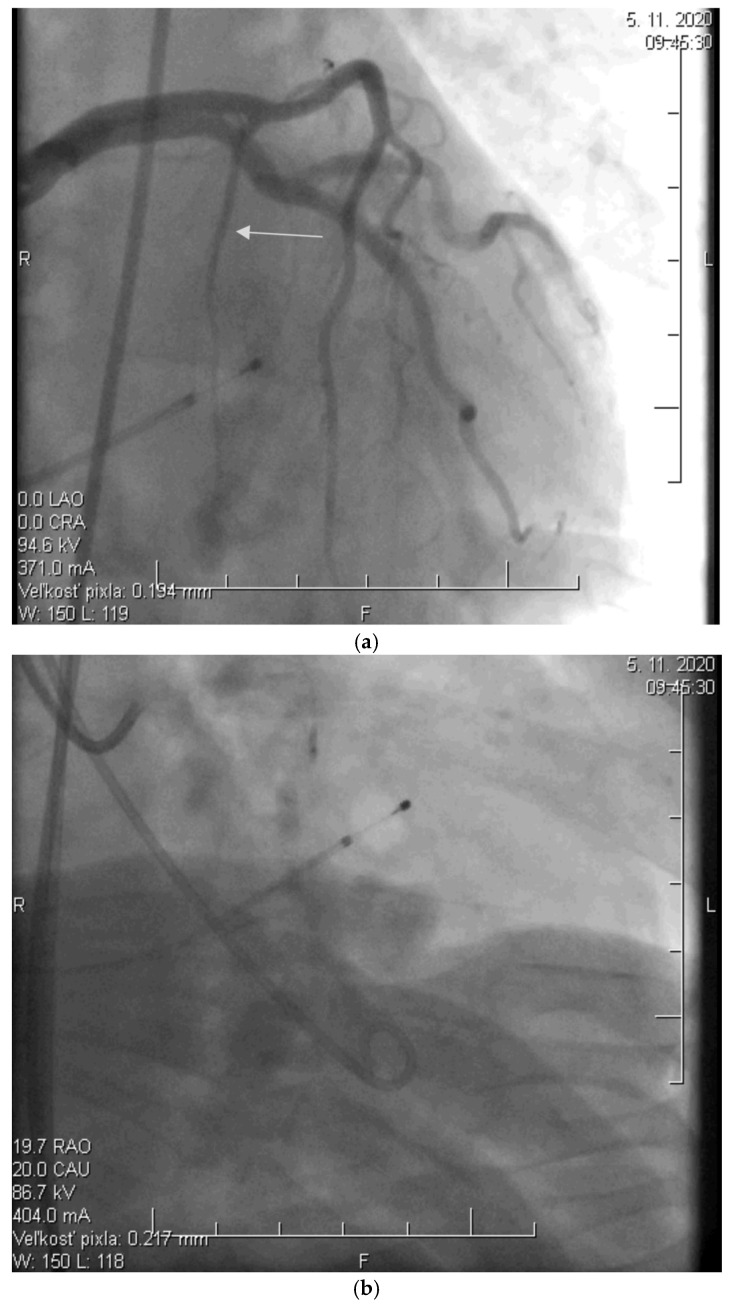

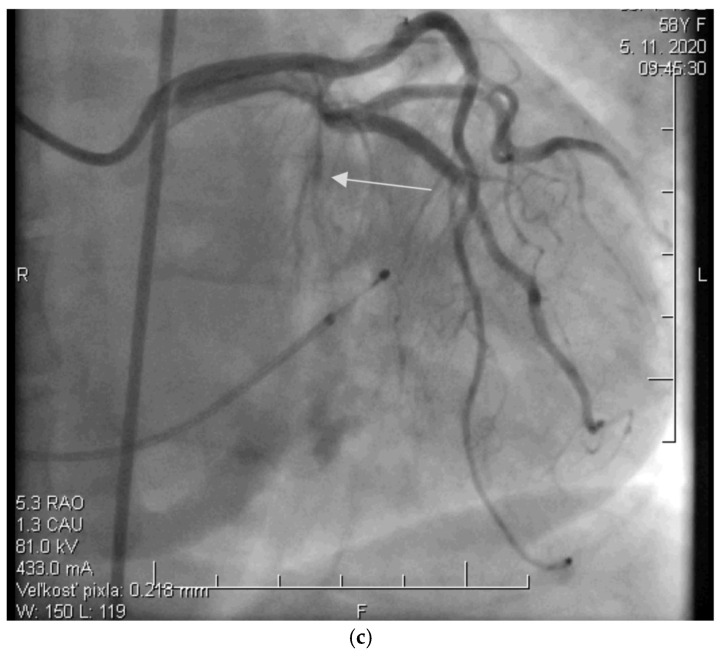
(**a**) Angiographic image of the left coronary artery showing a septal branch (arrow) (photo and copyright: T.P.). (**b**) Administration of 96% alcohol into the septal branch using an over-the-wire (OTW) balloon catheter (photo and copyright: T.P.). (**c**) Final effect of ASA with Closure of the Septal Branch (photo and copyright: T.P.).

Before the procedure, a temporary pacemaker was inserted into the RV via a venous approach to prevent potential periprocedural conduction disorders. After the pacemaker was placed, selective injection of the left coronary artery was performed via a transfemoral approach to assess the anatomy of its branches (Figure 2a). Using a pigtail catheter introduced into the LV, the pressure gradient between the LV apex and the aorta was measured at rest and after provocation with a ventricular extrasystole, Valsalva maneuver, or, less frequently, nitrate administration.

Subsequently, a specialized over-the-wire balloon catheter was inserted into the septal branch supplying the area contributing to the LVOT narrowing (most commonly the basal interventricular septum. The balloon catheter was inflated to occlude the lumen of this vessel, followed by the injection of an echocontrast agent and echocardiographic assessment of the perfusion area and localization of the selected septal branch (echo-guided approach). After identifying the appropriate septal branch, 96% alcohol in a volume of 2 mL was administered uniformly to all patients according to internal protocol (Figure 2b).

Ten minutes after the alcohol application, the balloon was deflated, and the residual pressure gradient between the LV apex and the aorta was measured at rest and after the aforementioned provocative maneuvers. To conclude the procedure, a selective injection of the LCA was performed to confirm the ablation of the septal branch and exclude potential alcohol leakage into other parts of the coronary circulation, which could cause the no-reflow phenomenon (Figure 2c).

A temporary pacemaker electrode was maintained for at least 24 h and extended in patients with bradyarrhythmias. In cases of an uncomplicated course, patients were monitored in the coronary care unit for 24–48 h before transfer to a general ward, with a minimum total hospitalization of 5 days, during which continuous cardiac rhythm monitoring was performed.

### 2.3. Echocardiographic Assessment

Each patient underwent a comprehensive transthoracic echocardiographic examination using the Philips EPIQ CVx device (Philips, Eindhoven, The Netherlands), which was performed by the same cardiologist. All parameters were recorded in accordance with the current recommendations using standard modalities such as 2D, M-mode, pulse, tissue, and continuous wave Doppler [10,11].

The following echocardiographic parameters of the RV were monitored at five distinct time points (pre-procedure (before ASA), 3 months post-procedure (early period), 1, 3 and 5 years post-procedure, respectively (later time interval)): wall thickness (RVWT), dimension of the proximal part of the outflow tract (RVOT_PROX_), maximum dimension of the base (RV_D_), end-diastolic volume (RV_EDV_), amplitude of lateral tricuspid annulus motion (TAPSE), and its maximum systolic velocity (S’), which were compared with standard indicators monitored in patients with HCM undergoing ASA, left ventricular ejection fraction (LVEF), maximum resting gradient in LVOT (LVOTG_MAX_), NYHA functional classification class, LV and left atrial end-diastolic dimension (LV_D_, LA_D_), interventricular septum dimension (IVS_D_), and LV posterior wall dimension (PW_D_). All echocardiographic variables were analyzed retrospectively from archived recordings. Each parameter was measured three times at each time point, and the average value was used for analysis. Measurements were performed offline and in accordance with existing guidelines for transthoracic echocardiography to ensure consistency and reliability.

### 2.4. Statistical Analysis

Changes in monitored parameters were analyzed using repeated-measures analysis of variance (ANOVA) followed by Tukey’s post hoc test. Pearson’s correlation coefficient was applied to assess associations between early and later parameter changes. All statistical tests were two-sided, with a significance threshold set at *p* < 0.05. The diagnostic accuracy of RVWT for predicting LVOT_G_ was assessed using Receiver Operating Characteristic (ROC) curve analysis, and the area under the curve (AUC) was calculated to evaluate discriminatory ability. Performance was interpreted according to established criteria: AUC 0.60–0.70 indicating poor, 0.70–0.80 fair, and 0.80–0.90 good accuracy. Statistical analyses were conducted using Prism version 9.3.0, and ROC analysis was performed with ROCFIT version 2.0.1.

## 3. Results

**Figure 3:**
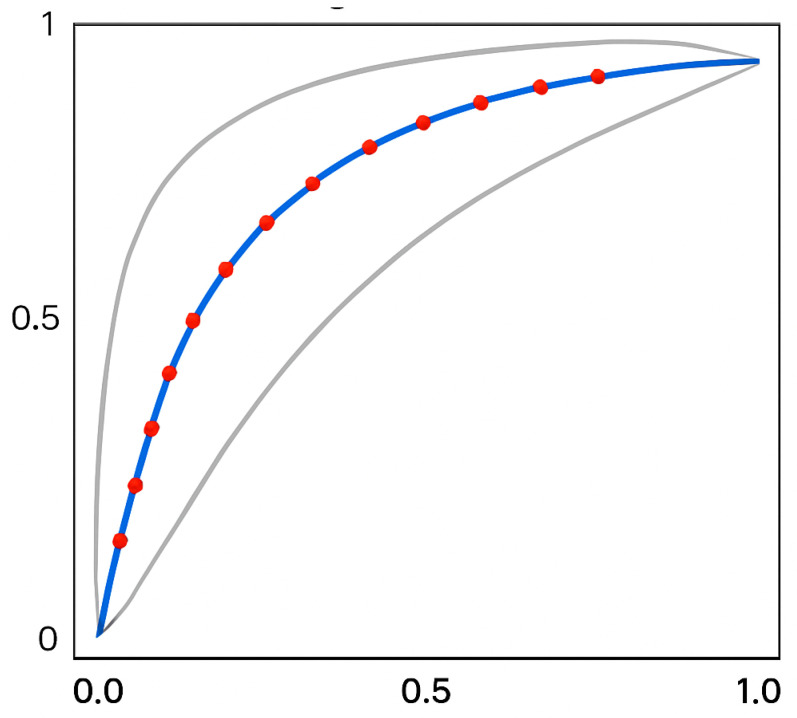
ROC curve illustrating the association between RVWT and LVOTG reduction following ASA (blue-red line—ROC curve; grey lines—95% confidence interval; X-axis—false positive rate; Y-axis—true positive rate; red dots—threshold points of RVWT; grey curves—95% confidence intervals) (photo and copyright: T.P.).

Echocardiographic findings are summarized in Table 1. Changes in RVWT measured by echocardiography at three months (r = 0.61; *p* < 0.001) and one year (r = 0.64; *p* < 0.001) post-procedure demonstrated significant correlations with baseline LVOT_G_ (Table 2). Significant associations were also observed during these intervals between RVWT and NYHA class (r = 0.52 and r = 0.55; both *p* < 0.01, Table 3) as well as between RVWT and LVD (r = 0.44 and r = 0.58; both *p* < 0.01, Table 4). ROC analysis identified a cut-off value of 1/RVWT at 0.5142 cm (equivalent to RVWT = 7.4 cm), yielding a sensitivity of 88% and specificity of 69.7%. The AUC was 0.869 (*p* < 0.05) (Figure 3). At years three and five, the correlations were no longer statistically significant (*p* = ns). No significant correlations were identified for other parameters.

Pharmacological therapy (betablockers or verapamil) at baseline follow-up was represented in 97% of patients. There was no significant change in medication at any time point (*p* = NS for all).

## 4. Discussion

To date, this investigation represents the first focused analysis of the prognostic value of echocardiographic right ventricular (RV) parameters in evaluating outcomes after alcohol septal ablation (ASA) in patients with hypertrophic cardiomyopathy (HCM). Previous studies have primarily compared RV function in HCM cohorts versus healthy controls, with inconsistent findings regarding the clinical impact of RV involvement. RV involvement, defined by increased wall thickness or impaired function, is present in a significant subset of HCM patients and is associated with more advanced disease, higher arrhythmic risk, and adverse clinical outcomes [12,13,14,15,16].

The present data indicate a significant association between right ventricular wall thickness (RVWT) and key clinical and echocardiographic outcomes, including left ventricular outflow tract gradient (LVOT_G_), New York Heart Association (NYHA) functional class, and left ventricular diameter (LV_D_) at both three months and one year following ASA. The most robust correlation was observed between baseline RVWT and subsequent LVOTG, suggesting that lower pre-procedural RVWT is linked to greater reductions in LVOTG after ASA. This relationship may reflect the interplay between RV hypertrophy and LV outflow tract obstruction, as well as the hemodynamic and structural remodeling that occurs following successful septal reduction therapy [17].

Notably, the cut-off value of RVWT associated with significant reductions in LVOTG was 7.4 mm, with high sensitivity and specificity. This threshold may serve as a useful marker for predicting procedural success and guiding patient selection for ASA. Importantly, RV systolic function was preserved in all monitored subjects, consistent with findings from other studies, which have shown that RV dysfunction is relatively uncommon in HCM but, when present, is associated with poor LV systolic function and worse prognosis [1,2].

The precise mechanisms underlying the relationship between RVWT and clinical indicators of ASA success remain incompletely understood. Some authors have proposed that loss of active RV outflow tract contractility and subsequent remodeling of specific RV segments after ASA may contribute to reductions in LVOT_G_, LV_D_, and NYHA class. Additionally, increased RV wall thickness has been correlated with higher LV mass, reduced LV longitudinal strain, and increased calculated sudden cardiac death risk scores, suggesting that biventricular involvement may reflect a more severe disease phenotype [18].

RV involvement in HCM is increasingly recognized as a clinically relevant factor, with recent meta-analyses demonstrating that RV systolic dysfunction independently predicts adverse outcomes, including all-cause mortality, heart failure hospitalization, and arrhythmic events [19,20]. The American College of Cardiology and American Heart Association recommend comprehensive echocardiographic assessment—including RV parameters—before and after septal reduction therapy to optimize procedural planning and monitor therapeutic response [21]. Advanced imaging modalities, such as cardiac magnetic resonance and speckle-tracking echocardiography, provide additional insights into RV structure and function, enabling more precise risk stratification and individualized management [12,15,19,22,23].

Overall, these results underscore the importance of integrating RV assessment into the routine evaluation of HCM patients undergoing ASA, as RV structural and functional parameters may provide additional prognostic information and help refine risk stratification and management strategies. Future research should focus on elucidating the mechanisms of RV remodeling in HCM, the genetic and molecular determinants of RV involvement, and the long-term impact of therapeutic interventions on RV function and clinical outcomes [24].

Right ventricular hypertrophy (RVH) in hypertrophic cardiomyopathy (HCM), defined as right ventricular wall thickness (RVWT) greater than 5 mm, can manifest in various regions of the right ventricle, including the free wall, basal interventricular septum, and apex. In many patients, hypertrophy is diffuse, affecting multiple segments of the RV [20]. There is a significant correlation between RVWT and left ventricular (LV) wall thickness, with greater RV hypertrophy paralleling more pronounced LV hypertrophy and mass [13]. This biventricular involvement is associated with more severe disease phenotypes, including higher left ventricular outflow tract gradients (LVOT_G_), increased risk of ventricular arrhythmias, and elevated calculated sudden cardiac death risk scores [23].

Patients with RV hypertrophy tend to experience more severe symptoms, such as exertional dyspnea and progressive heart failure, and demonstrate worse functional status as measured by NYHA class. RV systolic dysfunction, although less common, is independently associated with adverse outcomes, including increased risk of death and heart transplantation, particularly when accompanied by LV dysfunction and elevated pulmonary pressures [25]. The pathophysiology of RVH in HCM involves adaptive myocyte hypertrophy, fibrosis, and metabolic shifts, which may progress to RV failure if afterload remains chronically elevated [26,27].

Alcohol septal ablation (ASA) and other septal reduction therapies can lead to favorable remodeling of both ventricles, with reductions in LVOT_G_ and improvements in clinical status. However, the precise mechanisms linking RVWT to procedural success and long-term outcomes remain incompletely understood, and further prospective studies with larger cohorts are needed. The current consensus highlights the importance of comprehensive biventricular assessment using advanced imaging modalities, as RV involvement is a key determinant of prognosis and should inform risk stratification and management in HCM [3,7,15,28,29].

The main limitation of the study is its retrospective nature, arising from the fact this is a single-center study, which, given this diagnosis, inevitably leads to a relatively small sample size. Unfortunately, this proved to be particularly limiting when comparing data over a long-term period. The sample size was also influenced by the relatively strict inclusion and exclusion criteria. Lastly, there is an absence of definitive diagnostic confirmation via genetic testing; however, the diagnostic criteria were met for all patients included in the study.

It is also necessary to consider, when interpreting the results, that this is a non-randomized study conducted in a tertiary care center, meaning that the distribution of patients is not even in terms of baseline characteristics (e.g., functional status).

Finally, the absence of cardiac MRI assessment is another significant limitation, and it should be considered for inclusion in the dataset to strengthen the analysis in the future *(installation of equipment at our center is expected in December 2025)*.

## 5. Conclusions

Our study results suggest that RVWT measured prior to ASA may serve as a predictor of subsequent LVOT_G_ development and could potentially provide an early estimate of the long-term effects of ASA as soon as 3 months post-procedure, even in the context of continued myocardial remodeling. Further multicenter studies on a large group of patients are required to validate these findings.

## Figures and Tables

**Table 1:** Echocardiographic measurements of patients collected during the follow-up period.

	Before ASA	0–3 M		0–1 Y		0–3 Y		0–5 Y	
			*p*		*p*		*p*		*p*
RVWT, mm	7.1 ± 1.9	7.0 ± 1.9	<0.001	6.7 ± 1.8	<0.001	6.6 ± 1.6	<0.001	6.5 ± 1.6	<0.001
RVOT_PROX_, mm	29.2 ± 2.1	29.7 ± 2.0	ns	29.8 ± 1.8	ns	29.8 ± 2.3	ns	30.1 ± 2.2	ns
RV_D_, mm	39.0 ± 4.3	39.2 ± 4.2	<0.05	39.2 ± 4.3	<0.05	39.7 ± 4.0	ns	39.9 ± 3.8	ns
RV_EDV_, mL	143.7 ± 49.2	143.9 ± 49.0	ns	144.1 ± 48.5	ns	145.2 ± 47.9	ns	145.7 ± 48.6	ns
TAPSE, mm	23.3 ± 4.7	23.0 ± 4.4	ns	22.7 ± 4.3	ns	22.5 ± 3.9	ns	22.2 ± 4.0	ns
S’, mm	10.9 ± 1.4	10.7 ± 1.3	ns	10.9 ± 1.2	ns	10.5 ± 1.3	ns	10.4 ± 1.4	ns
LVOT_G_, mmHg	83.0 ± 32.8	19.9 ±12.7	<0.001	21.2 ± 13.8	<0.001	20.4 ± 15.5	<0.001	34.2 ± 30.9	<0.001
NYHA	2.8 ± 0.4	2.6 ± 0.3	<0.001	2.6 ± 0.5	<0.001	2.3 ± 0.6	<0.001	2.0 ± 0.5	<0.01
LV_D_, mm	40.3 ± 4.8	40.7 ± 4.9	<0.05	41.1 ± 4.7	<0.05	43.6 ± 4.9	<0.05	44.2 ± 5.3	<0.01
IVS_D_, mm	19.4 ± 3.5	19.1 ± 2.7	<0.001	16.4 ± 3.3	<0.001	14.8 ± 2.9	<0.001	14.6 ± 3.6	<0.001
PW_D_, mm	14.6 ± 3.3	14.2 ± 2.0	<0.01	13.7 ± 2.1	<0.01	13.2 ± 2.4	<0.01	13.0 ± 1.8	<0.001
LA_D_, mm	43.2 ± 6.9	43.0 ± 5.4	ns	42.7 ± 4.9	ns	42.4 ± 6.1	ns	43.8 ± 5.9	ns
LVEF, %	64.9 ± 5.5	60.6 ± 6.1	<0.01	61.8 ± 7.2	<0.01	64.1 ± 7.4	ns	62.3 ± 7.0	ns

IVS_D_—interventricular septum dimension; LA_D_—left atrial dimension; LV_D_—left ventricular end-diastolic dimension; LVEF—left ventricular ejection fraction; LVOT_G_—left ventricular outflow tract gradient; M—months; ns—not significant; NYHA—New York Heart Association Functional Classification; PW_D_—posterior wall dimension; RV_D_—right ventricular end-diastolic dimension; RV_EDV_—right ventricular end-diastolic volume; RVOT_PROX_—right ventricular outflow tract at proximal; RVWT—right ventricular wall thickness; S’—peak systolic velocity of the tricuspid annulus; TAPSE—tricuspid annular plane systolic excursion; Y—years.

**Table 2:** Correlations between right ventricular echocardiographic parameters and LVOT_G_ values in patients undergoing ASA treatment.

	0–3 M		0–1 Y		0–3 Y		0–5 Y	
	r	*p*	r	*p*	r	*p*	r	*p*
RVWT, mm	0.62	<0.001	0.63	<0.001	0.52	ns	0.67	ns
RVOT_PROX_, mm	−0.71	ns	−0.72	ns	−0.80	ns	−0.81	ns
RV_D_, mm	−0.48	ns	−0.27	ns	−0.52	ns	−0.12	ns
RV_EDV_, mL	−0.61	ns	−0.78	ns	−0.71	ns	−0.28	ns
TAPSE, mm	−0.63	ns	−0.77	ns	−0.59	ns	−0.56	ns
S’, mm	−0.32	ns	−0.51	ns	−0.22	ns	−0.28	ns

M—months; ns—not significant; r—correlation coefficient; RV_D_—right ventricular end-diastolic dimension; RVEDV—right ventricular end-diastolic volume; RVOT_PROX_—right ventricular outflow tract at proximal; RVWT—right ventricular wall thickness; S’—peak systolic velocity of the tricuspid annulus; TAPSE—tricuspid annular plane systolic excursion; Y—years.

**Table 3:** Correlations between right ventricular echocardiographic parameters and NYHA functional class in patients treated with ASA.

	0–3 M		0–1 Y		0–3 Y		0–5 Y	
	r	*p*	r	*p*	r	*p*	r	*p*
RVWT, mm	0.53	<0.01	0.54	<0.01	0.61	ns	0.69	ns
RVOT_PROX_, mm	0.65	ns	0.68	ns	0.68	ns	0.78	ns
RV_D_, mm	0.52	ns	0.24	ns	0.57	ns	0.46	ns
RV_EDV_, mL	0.64	ns	0.76	ns	0.64	ns	0.52	ns
TAPSE, mm	0.68	ns	0.79	ns	0.51	ns	0.66	ns
S’, mm	0.40	ns	0.46	ns	0.39	ns	0.42	ns

M—months; ns—not significant; r—correlation coefficient; RV_D_—right ventricular end-diastolic dimension; RVEDV—right ventricular end-diastolic volume; RVOT_PROX_—right ventricular outflow tract at proximal; RVWT—right ventricular wall thickness; S’—peak systolic velocity of the tricuspid annulus; TAPSE—tricuspid annular plane systolic excursion; Y—years.

**Table 4:** Correlations between right ventricular echocardiographic parameters and LV_D_ values in patients treated with ASA.

	0–3 M		0–1 Y		0–3 Y		0–5 Y	
	r	*p*	r	*p*	r	*p*	r	*p*
RVWT, mm	0.43	<0.01	0.59	<0.01	0.68	ns	0.64	ns
RVOT_PROX_, mm	0.58	ns	0.72	ns	0.64	ns	0.72	ns
RV_D_, mm	0.63	ns	0.27	ns	0.61	ns	0.51	ns
RV_EDV_, mL	0.59	ns	0.82	ns	0.62	ns	0.58	ns
TAPSE, mm	0.63	ns	0.77	ns	0.59	ns	0.72	ns
S’, mm	0.44	ns	0.53	ns	0.48	ns	0.51	ns

M—months; ns—not significant; r—correlation coefficient; RV_D_—right ventricular end-diastolic dimension; RVEDV—right ventricular end-diastolic volume; RVOT_PROX_—right ventricular outflow tract at proximal; RVWT—right ventricular wall thickness; S’—peak systolic velocity of the tricuspid annulus; TAPSE—tricuspid annular plane systolic excursion; Y—years.

## Data Availability

The datasets generated or analyzed during this study are available from the corresponding author upon reasonable request.
